# The dignity of burn patients: a qualitative descriptive study of nurses, family caregivers, and patients

**DOI:** 10.1186/s12912-021-00725-w

**Published:** 2021-10-22

**Authors:** Banafsheh Tehranineshat, Mahnaz Rakhshan, Camellia Torabizadeh, Mohammad Fararouei, Mark Gillespie

**Affiliations:** 1 Present Address: Community Based Psychiatric Care Research Center, Department of Nursing, School of Nursing and Midwifery, Shiraz University of Medical Sciences, Shiraz, Iran; 2 Department of Epidemiology, School of Public Health, Shiraz University of Medical Sciences, Shiraz, Iran; 3 School of Health Nursing and Midwifery, University of the West of Scotland, Paisley, Scotland UK

**Keywords:** Burns, Patients, Human dignity, Respect, Nursing, Family caregivers, Qualitative research

## Abstract

**Background:**

As an ethical principle, showing respect for human dignity is a professional duty of all nurses. The aggressive nature of severe burn injuries makes it hard to respect the existential values and dignity of burn patients. However, only a few studies have been conducted on the preservation of the dignity of burn patients. The purpose of this study is to identify and describe burn patients’ dignity as perceived by nurses, family caregivers, and burn patients.

**Methods:**

The present study has a descriptive, qualitative research design. Nurses, family caregivers and patients in the biggest burns hospital in the south of Iran were selected via purposeful sampling from October 2017 to August 2018 (*n* = 25). Data were collected using semi-structured, in-depth, individual interviews. Thereafter, data analysis was performed through conventional content analysis.

**Results:**

Three main themes were extracted from the information obtained in the interviews: empathic communication, showing respect, and providing comprehensive support**.**

**Conclusion:**

The care provided to burn patients should be combined with effective communication, spending time with them, and attending to their repetitive requests, so that they can freely express their feelings and concerns. In addition, the patients’ human values and beliefs should be respected and all aspects of their existence should be taken into account to preserve their dignity. Workshops designed based on the findings of the present study can help with improving the quality of burn nursing care.

**Supplementary Information:**

The online version contains supplementary material available at 10.1186/s12912-021-00725-w.

## Background

Burn injury is known as one of the leading causes of morbidity and mortality worldwide [1]. Individuals from all age groups and various socioeconomic levels are vulnerable to burn injuries [2]. The aggressive nature of severe burns and the heavy burden of dealing with the consequences, including impaired physical functions, disfigurement, and psychological distress, can threaten the victims’ human dignity [3, 4].

A basic concept in nursing ethics, dignity is comprised of (intrinsic) personhood, (rational/behavioral) sociability, respect, and independence. The nurses in burns departments should provide care combined with respect [5]—it is part of their professional duties to preserve their patients’ dignity as every patient is entitled to be treated with respect [6]. Nurses are expected to respect their patients when they are caring for them regardless of their gender, age, personality, health conditions, or financial status [7].

Protection of dignity is associated with increased patient satisfaction and self-esteem, reduced length of hospital stay, enhanced ability of patients to cope with their illness, and giving patients the sense that their lives matter [8]. Respect for human dignity enhances caregivers’ motivation to provide high quality care, which consequently results in an effective relationship between patients and caregivers [9]. In contrast, violation of patient dignity leads to patients’ psychological and spiritual distress, reduced motivation to survive, and deterioration of their physical and mental health conditions [10].

In the literature, there are several studies on the nature of dignity of different groups of patients [11–14]. According to these studies, patients see the following as the main aspects of their dignity: feeling that they are in control and have autonomy, being respected, not being exposed, feeling that their information is kept confidential and their needs are met [15], having their privacy maintained, feeling that their values are respected, and receiving adequate support [16, 17].

However, our knowledge of the ethical aspects of caring for burn patients is still limited [18], and despite the serious psychological, social, cultural, and spiritual consequences of burn injuries, the dignity of burn patients has not been explored in any studies yet [19].

Dignity is a concept that should be defined based on cultural contexts and physical environments [4]. Human dignity is an ultimate and irreducible entity which is greatly superior to other human values. This view on the concept of dignity is compatible with religious points of view in this regard [20]. Correspondingly, the dominant religion in Iran is Islam, and the Islamic culture attaches great importance to showing respect for individuals’ rights [21].

Clarifying the meaning of burn patients’ dignity is crucial to fully respecting and preserving the dignity of this group. Therefore, it is necessary to establish the meaning of dignity as well as its dimensions in this group of patients. Due to the abstract nature and complexity of the concept of dignity, a qualitative study is required to achieve a clear and in-depth knowledge of this concept [8, 22]. Besides, a qualitative approach helps to identify the problems related to burn injuries and to achieve an extensive understanding of the issues of burn patients [2]. Research on burn patients’ dignity can help with identifying the effective factors in maintaining the dignity of burn patients, and, consequently, providing high quality care. Thus, the findings of the present study can help healthcare administrators and caregivers in providing a supportive and empowering environment where burn patients’ rights and dignity are maintained. The aim of the present study is to identify and describe burn patients’ dignity based on the experiences of nurses, family caregivers, and burn patients.

## Specific objectives

1. What are the experiences of nurses, family caregivers, and burn patients regarding burn patients’ dignity?
2. Based on their experiences, how do nurses, family caregivers, and burn patients describe burn patients’ dignity?

## Methods

### Study design

The present study is a descriptive qualitative work of research conducted using the content analysis approach [23]*.*

### Participants and sampling method

A total of 14 nurses, 6 family caregivers, and 5 patients were selected via purposeful sampling. The study lasted from October 2017 to August 2018. After making arrangements with the hospital matron, the first researcher (BT) visited different departments of the hospital on different days and different work shifts (morning, afternoon, night). After explaining the research objectives to the heads of each department, she asked them to give her the names of the nurses, patients and family caregivers who met the inclusion criteria for individual interviews [24]. The researcher chose participants who had rich knowledge and experience of burn patients’ dignity to obtain a comprehensive insight into the research questions [25]. The sample size was determined so as to ensure data saturation. Data saturation has been reached when no new categories can be extracted from the collected data and all the categories are saturated in terms of feature and dimension [26]. The inclusion criteria for the nurses were as follows: having at least a bachelor’s degree, a minimum of 6 months’ work experience, experience of caring for a second-degree or a third-degree burn patient, and not being fatigued due to work overload in the hospital. The inclusion criteria for the family caregivers were: being 18 years old or above, being a member of the patient’s family, e.g. a spouse, child, sibling, or a friend of the patient, having no psychological or metabolic disorder to their knowledge, not being on any medication affecting the mind, not suffering from physical or psychological fatigue due to caring for their patients, being mentally and physically fit to be interviewed, and having experience of caring for a second-degree or a third-degree burn patient. The inclusion criteria for the inpatients were as follows: being over 18 years old, having second or third degree burns; having been burned by accident (not intentionally), having no confirmed psychological disorder or mental retardation, not being addicted to LSDs, drugs, or alcoholic drinks before or after receiving their burn injuries.

Being willing to participate in this study, being able to provide meaningful and rich information on the subject of the study, and speaking and understanding Farsi were essential to the inclusion of all the participants.

### Research settings

The research context was Amir-Al-Moemenin Burn and Plastic Surgery Hospital which is the largest and most advanced burn care and treatment hospital in the south of Iran. This hospital offers all kinds of medical services related to burn injuries. The research setting comprised of all the units of the hospital, including the men’s burns unit, the women’s burns unit, the emergency department, the plastic surgery department, and ICU. All the units of this educational hospital provide specialized care in the field of burns, rehabilitation, and plastic surgery. The additional special care services offered by this facility are the following: pain-free dressing, laser therapy, amnion dressing, and skin graft dressing.

### Data collection method

Data were collected through face-to-face, semi-structured interviews. Overall, 27 interviews were conducted with 14 nurses, 6 family caregivers, and 5 inpatients. Two of the patients were interviewed twice.

As for the research team, the first three authors had a PhD in nursing and about 15–25 years of clinical training experience. All the researchers were trained and skilled in qualitative research. The present study was conducted to fill the gap in the existing literature on the dignity of burn patients.

Due to lack of a specific protocol which recognizes the dignity of burn patients (in hospitals) in Iran, a comparative approach was adopted to identify qualified participants. The interviews were conducted and recorded by the first researcher (BT) in the nurses’ break room or at the conference hall of the hospital, with the permission of the ward’s head nurse. During these interviews, none of the other staff members were allowed to enter the room/conference hall without permission.

In order to focus on the dimensions of burn patients’ dignity, the first author (BT) had initially interviewed a nurse, a family caregiver and a burn patient (not actual participants) in order to finalize the interview guideline. Therefore, this interview guideline was oriented around the subjects’ perceptions and definitions of burn patients’ dignity, as well as their past and present experiences. Afterward, in order to encourage the participants to answer her questions openly and sincerely, the first author (BT) informed the participants that she was a university faculty member, all information would remain confidential and anonymous, and participation was on a voluntary basis. Before each interview, the objectives and method of the study were explained to the participants and they were told that the sponsorship was non-commercial.

The interviews started with a general question-“what does the concept of burn patients’ dignity mean to you?”-to allow the participants to describe their understanding and experiences completely. Thereafter, based on the participants’ responses, more specific questions were asked to gather information related to the objectives of the study more directly (additional file 1). To obtain more clear answers, the interviewer used open-ended questions, such as “What do you mean by that?”, “Can you explain further?”, and “Can you give an example?”

The participants’ voices were recorded using a Sony Voice Recorder ICD-TX650. The duration of the interviews varied from 45 to 70 min. During the interviews, the participants’ nonverbal communication was also noted.

### Data analysis

As data were being collected, they were analyzed according to Graneheim and Lundman’s (2004) approach to qualitative content analysis [27]. Accordingly, immediately after conducting each interview, the first author (BT) transcribed the interview and read and re-read the transcript. Subsequently, after obtaining a general understanding of the content, she executed an inductive analysis of the information. At the reading stage, important paragraphs were carefully read line by line. Words, sentences, or paragraphs that were significant regarding burn patients’ dignity were designated as semantic units. Thereafter, a code was assigned to each key paragraph or phrase.

Subsequently, the second author (MR) reviewed the transcripts and then verified the semantic units and open codes. Possible disagreements over the semantic units and codes were resolved in a meeting attended by all the four researchers (BT, MR, CT and MF). Categories were then generated based on the similarities and differences among the codes. To ensure maximum strength of the codes, all the categories were revised and then compared with the collected data. Next, in several meetings, the research team members (BT, MR, CT and MF) extracted the themes by careful and in-depth contemplation and comparing the categories with each other.

During the data collection and analysis processes, the researchers applied bracketing. Bracketing is a method commonly used in qualitative research in order to mitigate the potentially undesirable effects of preconceptions that may cause bias in research [27]. Hence, the researchers attempted to ignore their own knowledge, beliefs, values, and experiences to describe the participants’ points of view on the concept of burn patients’ dignity accurately and objectively. The researchers did not form any judgment on the data and accepted them as they were. Data analysis was managed using MAXQDA 2007.

### Trustworthiness

The accuracy and trustworthiness of the data were tested according to Lincoln and Guba’s criteria [28]. To ensure the credibility of the data, the researchers applied prolonged engagement, member checking, and peer debriefing. Before conducting the study, the first researcher (BT) was present in the wards as a nurse instructor and had regular interactions with the participants during the course of the study. Afterward, the collected data were reviewed with the participants (nurses, family caregivers, and patients) and then examined via triangulation (nurses, family caregivers, and patients from different genders and age groups) and maximum variation sampling based on contrasting evidence. Furthermore, in order to confirm the dependability and conformability of the obtained data, the researchers had a panel of experts examine the transcripts, extracted codes, and categories.

To test the transferability of the findings to other similar groups and settings, the researchers asked several burn care nurses, family caregivers, and burn patients who had not participated in the study to evaluate the degree to which the results reflected their own experiences. The researchers also requested feedback from the experts and the participants (including peer review and revision of the manuscripts by the participants in addition to professors and colleagues who were familiar with qualitative research). The collected information not only confirmed the reliability of the findings of the study, but also provided the researchers with further rich experiences and complementary views which were considered in data analysis.

## Results

Twenty-five subjects participated in the present study. The age ranges of the nurses, family caregivers, and patients were 28–54, 22–41, and 24–48 years old, respectively. The work experience of the nurses ranged from 2 to 28 years, with the average of 13.71 ± 9.78 years. The demographic characteristics of the participants are shown in Table 1.

**Table 1:** The demographic characteristics of the participants

No	Participant	Position	Marital Status	Education Level
P1	Nurse	Staff nurse	Married	Bachelor
P2	Nurse	Staff nurse	Single	Bachelor
P3	Nurse	Matron	Single	Bachelor
P4	Nurse	Head nurse	Married	Bachelor
P5	Nurse	Staff nurse	Married	Bachelor
P6	Nurse	Supervisor	Single	Master
P7	Nurse	Staff nurse	Single	Bachelor
P8	Nurse	Supervisor	Married	Master
P9	Nurse	Staff nurse	Single	Bachelor
P10	Nurse	Staff nurse	Single	Bachelor
P11	Nurse	Staff nurse	Married	Bachelor
P12	Nurse	Staff nurse	Married	Bachelor
P13	Nurse	Staff nurse	Married	Bachelor
P14	Nurse	Staff nurse	Single	Bachelor
P15	Family Caregiver	Housewife	Single	Diploma
P16	Family Caregiver	Housewife	Single	Diploma
P17	Family Caregiver	Employee	Married	Bachelor
P18	Family Caregiver	Self-employed	Married	Illiterate
P19	Family Caregiver	Self-employed	Single	Diploma
P20	Family Caregiver	Housewife	Married	Illiterate
P21	Patient	Employee	Married	Bachelor
P22	Patient	Housewife	Single	Diploma
P23	Patient	Self-employed	Married	Diploma
P24	Patient	Housewife	Married	Illiterate
P25	Patient	Self-employed	Single	Diploma

Data analysis yielded three main themes: empathic communication, showing respect, and providing comprehensive support. The themes and their categories are shown in Table 2.

**Table 2:** The themes and categories of this study

Themes	Category
Empathic communication	Empathy
	Effective communication
	Dedicating time to the patients
Showing respect	Respect for human equality
	Respect for patient autonomy
	Respect for beliefs and values
	Respect for sexual privacy
	Avoidance of pity
Comprehensive support	Pain relief
	Psychological support
	Social support

### Empathic communication

One of the most important findings of the study, was empathic communication, a dimension referred to by all the participants. From the participants’ point of view, when an emotional connection is established through empathy, the patients can share their problems with their nurses. By treating their patients with kindness and dedicating time to them, nurses can build emotional intimacy with patients and also impart a sense of comfort, trust, and respect to them. This theme is comprised of 3 categories: empathy, effective communication, and dedicating time to patients.

#### Empathy

From the viewpoint of the caregivers, empathy is of great importance in burns departments due to the special nature of burn injuries and the difficult conditions of the patients. Burn patients’ health and quality of life are usually very adversely affected by their injuries. Because they are usually hospitalized for long periods, nurses have more time to be empathetic to the patients. This type of relationship helps nurses understand their patients better and, consequently, support them more effectively. Every burn patient needs physical and psychosocial care according to his/her specific needs. Regarding showing empathy for burn patients, one participant stated that:

"A burn patient needs a lot of empathy and emotional closeness; in fact, empathy is complementary to our treatment. No matter how good the care we provide is, it is not going to work without empathy … "(P1).

From the nurses’ point of view, burn patients are less likely to complain of pain when they realize that their nurses empathize with them, so the nurses can help the patients more effectively.

"When I imagine myself in his [a burn patient's] position, it helps me a lot in understanding his issues and concerns, and then I can help him more ..." (P6).

#### Effective communication

According to the patients who participated in this study, dignified care is possible through proper and effective communication. It was indicated that only when nurses talk to patients and use body language are patients encouraged to feel free to ask their questions and express their concerns and requests.

According to the participants, nonverbal forms of communication are an indispensable part of dignified care and reflect nurses’ feelings and perceptions of patients. Listening well and making eye contact with patients are signs of valuing patients and making an effort to understand their concerns. With respect to the significance of nonverbal communication skills, one patient stated:

“My nurse listened to me well … she made eye contact with me every time she wanted to talk with me … her behavior made me feel valued …” (P22)

From the participants’ point of view, dignified care is a function of nurses’ use of verbal and nonverbal communication which enables nurses to identify the best strategy to manage each patient’s pain and other issues, as well as to help them cope with their current situation.

The patients believed that caregivers’ use of kind words during care, addressing the patients by such terms as “dear,” and appreciation of the patients’ patience at the end of painful procedures are among the communication skills which help preserve patients’ dignity during their hospital stay.

“There was a nurse here who always used kind words like “dear” and “sweetheart” to address the patients whenever she wanted to talk to them ... Her kind words showed that she cared about the patients …” (P21).

The nurses believed that burn patients can express their concerns more freely when nurses establish an effective non-verbal communication with them.

#### Dedicating time to the patients

According to the interviews with the participants, burn patients feel more intimate and talk more freely when their caregivers spend time with them. This also motivates patients to cooperate in their treatment process. On the other hand, nurses’ failure to spend quality time with their patients creates feelings of worthlessness and indignity in the latter.

"Sometimes, I talk to my patients for a long time about their problems as well as my own experiences .... When the patients realize how much I want to spend my time with them, they feel valued … " (P10).

Also, referring to the helplessness and incapacity of burn patients, the participants deemed dedicating adequate time to the patients and dealing with their frequent requests as essential to maintaining burn patients’ dignity.

“These patients make a lot of requests; they’re always calling their nurses … . The nurses who devote time to their patients, pay attention to their repeated request … they are actually showing that they value their patients … ” (P 8).

### Showing respect

From the caregivers’ viewpoint, the traumatic experience of suffering a burn, painful hospital treatments, body dysfunctions, and expressions of pity from others cause burn patients to feel that they are not treated with dignity. Nurses can preserve the dignity of burn patients while caring for them by showing respect for their identity and autonomy and by involving the patients in their treatment plans. The theme of showing respect consists of the categories of respect for human equality, respect for patient autonomy, respect for beliefs and values, respect for sexual privacy, and avoidance of pity.

#### Respect for human equality

One of the major challenges of preserving the dignity of burn patients while providing care to them is cultural differences. The population of Iran is comprised of different races and ethnic groups. The majority of the population in Iran is Fars, but caregivers are likely to meet burn patients from Baloch, Azeri, Turk, Kurd, Lur, or Arab minorities. Patients’ cultural, ethnic, and social backgrounds can affect the sense of value of the patients as well as their families.

The participants of the present study stated that all patients are equal. Accordingly, caregivers must preserve the dignity of all patients, regardless of their gender, religion, race, ethnicity, economic status, and social class. One of the nurses stated that:

“Most of the patients in this hospital are from the poor classes of the society, but we give them all the services they need, regardless of their ethnicity or social class … ” (P9).

A family caregiver said:

“When a nurse provides care to all her burn patients fairly and equally, no matter what the cause of their injuries, self-immolation or an accident, this reflects preservation of human dignity...” (P15).

#### Respect for patient autonomy

Another gesture that demonstrates respect for the dignity of burn patients is maintaining patient autonomy. From the participants’ points of view, patients should be free to choose their doctors and to reject or accept any recommended medical procedure. It is also important that patients must be given the chance to make informed decisions. By providing patients with the necessary information on different types of treatments and their possible side effects, nurses can facilitate patients’ informed interactive participation in the decision making process around their care. The participants regarded showing respect for patients’ freedom of choice and the right to participate in their care as a sign of respecting patients’ autonomy and, by expansion, their dignity.

"It is the worst kind of disrespect to give no explanation to a burn patient. I involve my patients in their treatment plans, which encourages them to cooperate with me..." (P5).

In the present study, the nurses provided their patients with educational tools, informed them about their role in their own care process, and provided cooperative follow-up to help their patients perform their daily activities and regain their kinesthetic abilities, which inspired their patients with a sense of value. To maintain the dignity of burn patients, the participating caregivers tried to inform their patients about their treatment and showed them pictures of healed burn injuries to help them in making better decisions.

“Some patients resist skin graft. I usually use educational pamphlets to explain their situation to them and some photos of burn injuries which have healed after skin graft … I talk to them. … In fact, by informing the patients, I help them decide how they want to continue their treatment … ” (P1).

The patients’ experiences showed that the members of treatment teams try to encourage patients to play an active role in making decisions about their treatment process.

“My nurses explained the purpose of everything they did for me, like the replacement of the dressing of my wounds, my medication and its side effects … they informed me of the possible side effects of my surgery and by raising my awareness, they gave me freedom of choice … all of which made me feel valued … ” (P23)

#### Respect for beliefs and values

According to the participants, burn patients tend to become interested in mystical, spiritual, and religious practices after suffering burn injuries. By providing prayer services, arranging meetings with a priest for them, and providing access to prayer books, caregivers can show respect for the patients’ dignity. One participant said:

“Many patients here believe in praying. To respect the dignity of these patients, we've provided them with a bookshelf full of prayer books and this has really helped their recovery process ...” (P5).

#### Respect for sexual privacy

According to the participants, showing respect for the sexual privacy of burn patients is essential to maintaining their dignity. The participants’ experiences show that receiving care from a nurse of the opposite-gender threatens the patients’ dignity.

Every morning, they change my dressing … . It makes me uncomfortable when a female nurse washes me … nurses should wash and dress patients who are the same sex as themselves … (P25).

#### Avoidance of pity

The family caregivers were found to have experienced pitying looks and words directed at their patients, which had undermined the dignity of their patients as they believed.

The caregivers in the present study mentioned that although burn injuries can cause serious changes in burn patients’ physical functions and appearances, the patients should be treated as ordinary people and the treatment team should avoid pitying behaviors. However, the experiences of the interviewed family caregivers showed that they could detect pity in other nurses’ behaviors and expressions of emotions, which made it harder for their patients to tolerate their special conditions. The patients were not only forced to tolerate the pain and physical problems caused by their injuries, but also had to bear the pitying attitude of the nurses around them. This was regarded as disrespect for the patients’ dignity.

“Burns patients do not need pity, and yet some nurses pity them. For instance, they say things like “Oh, poor thing!”... This can make the patient feel disrespected ...” (P5).

The family caregivers declared that they expected more professional and systematic behaviors from the treatment teams compared to others. They expected the members of treatment teams, who had received education and training in this area, to have good communication skills and to be able to express empathy with no expression of undue pity which could add to patients’ psychological concerns. The family caregivers’ experiences underline the importance of avoiding pitying behaviors in maintaining patient dignity.

“Patients with burn injuries need to be treated with respect … They [nurses] should not give the impression that our patients would never recover … Being pitied by others is a sign of disrespect for these patients … ” (P18)

### Comprehensive support

The findings of the study indicated that comprehensive support is an important contributory factor in maintaining burn patients’ dignity. Comprehensive support was found to consist of the following categories: pain relief, psychological support, and social support.

#### Pain relief

The majority of the interviewed nurses described pain as the primary physical cause of complaint amongst burn patients. It was mentioned that any slight movement or the implementation of various treatment procedures can increase patients’ pain. The participants stated that, since burn patients may suffer from multiple injuries and functional disabilities, relieving the intensity of their pain using different methods and regular evaluation of the efficacy of the measures taken to manage their pain demonstrate respect for the dignity of the patients. According to a nurse:

“We have lots of patients who have sustained severe injuries to their organs or suffered spinal cord injuries due to electrocution, and most of them are constantly complaining of pain. Having their wound dressings replaced is a really painful experience. We use partial anesthesia when we want to replace a patient's dressing. After performing the procedure, if the patient is still in pain, painkillers are administered to them. We may use non-pharmacological interventions, too ...” (P12).

#### Psychological support

From the perspective of the participants, burn injuries not only affect the patients’ bodies, but also affect the psychological health of the patients and their families. Burn patients desperately need the support of the people around them. Because the treatment procedures are relatively long and can be very painful, they are implemented at several stages during which time the patients may suffer depression, isolation, anxiety, humiliation, and loss of dignity. One of the most important aspects of nursing burn patients is providing them with the emotional and psychological support required to preserve their dignity and to facilitate their recovery.

“Once, we had a patient who was seriously ill and her husband did not allow her family to visit her. I did my best to convince her husband to change his mind .... The patient finally saw her family, and afterward she just felt very valued and then recovered more quickly … ” (P1).

Based on the patients’ experiences, paying attention to the emotional and psychological needs of burn patients signifies respect for their dignity. According to a patient:

“I missed my son so much during my treatment process … my nurse made arrangements with my family … they brought my child to the entrance of the hospital … my nurse put me in a wheelchair and took me to the entrance so I could see my child … ” (P24).

Based on the experiences of the caregivers of burn patients, it is important to identify the origin of the patients’ mental distress and then make an effort to resolve it toward maintaining their dignity.

“Here, we have patients who are scared of death. Family visits can help them a lot to cope with this fear. For these patients, we usually assign a round-the-clock companion … ” (P4).

Other measures which can help with preserving burn patients’ dignity are the following: evaluating their mental and emotional well-being, providing them with counseling services, and promoting collaboration between the members of the burn care team and the patients’ families.

“Drug Addiction, family problems, and fear of alienation from the family and society are known as the leading psychological problems of burn patients. So, we should attempt to reduce the patients' psychological distress by raising their awareness, arranging counseling sessions for the patients and their families, removing the gaps between the patients and their family members, and facilitating cooperation between the treatment team and the patients' families ...” (P11).

Another nurse stated:

“After discharging a patient, we introduce him or her to a counseling center where they and their families can receive counseling services ...” (P13).

#### Social support

According to the participants’ experiences, burn patients encounter various social issues, like social stigma, which can cause them to quit their studies or jobs or get divorced. These problems pose a threat to the social dignity of burn patients. The category of social support consists of two sub-categories: avoidance of stigma and financial support from family and charity.

#### Avoidance of stigma

This sub-category concerns people’s wrong beliefs about, criticism of, and ostracizing burn patients. The participants’ experiences showed that most families believe that their patients with burn injuries have lost many of their former capabilities. Moreover, some individuals have wrong ideas about the causes of burn victims’ injuries (they considered self-immolation or punishment by the patients’ parents to be the cause) which results in their spreading false rumors about the patients’ source of injuries. According to one of the family caregivers:

“Many of our relatives thought that my sister had lost her fertility and could never have a baby … ” (P20).

The patients mentioned that they were criticized by their families and friends and also blamed for what had happened to them. In some cases, the patients’ families, especially spouses, were criticized for their manner of dealing with the patients.

#### Financial support from family and charity

Based on the participants’ experiences, the treatment of burn patients is often a lengthy process accompanied by anxiety and pain, which causes financial distress in addition to health complications and physical problems. The treatment of and follow-up care for burn injuries is very costly and patients often have to ask their families or relatives for financial help. When such requests are granted, patients feel valued and dignified.

“Financially speaking, my family has helped me a great deal. Right now, they have already paid for my surgery and medication … they say they would do anything to see me completely recovered … . Well, it makes me feel valued to know that I am important to my family … ” (P22).

According to the participants, another issue that is a threat to the dignity of burn patients is the social problems which arise after the event. Divorce, disintegration of the family, and stigmatization, which often causes patients to quit their studies or resign from their jobs, are among the social harms that adversely affect the social dignity of burn patients. One of the participating nurses pointed out:

“I know many patients who decided to quit their jobs due to the inappropriate behavior of the people around them … ” (P12).

From the viewpoint of the caregivers interviewed in the present study, the financial support provided by insurance companies, charity institutes, and social welfare organizations is quite limited, so burn patients view their nurses and family caregivers as their main sources of support. Therefore, nurses and social workers can preserve the patients’ social dignity by helping them learn job skills and find new jobs and by referring them to charity centers to help them with paying their treatment costs.

“We had a patient here whose spouse divorced her because of her facial burn marks and then she had no source of income and came to us. Here, the personnel try to help these people any way they can, for example by introducing them to charities, finding jobs for them, and paying for their medications” (P10).

## Discussion

The results of the present study show that nurses’, family caregivers’, and burn patients’ perceptions of burn patients’ dignity fall into three main themes: empathic communication, showing respect, and providing comprehensive support. According to the data obtained from the interviews, empathizing with burn patients in an effective relationship and dedicating time to them could make the patients feel dignified. In such an atmosphere, nurses can preserve the dignity of the patients by showing respect for the patients’ intrinsic values and autonomy and also by attempting to meet their emotional, psychological, and social needs.

In the present study, the theme of empathic communications was found to be comprised of the following categories: empathy, effective communication, and dedicating time to patients. Nurses should attempt to form a deep human and spiritual connection with their patients so that they can imagine themselves in their situation to preserve their dignity properly. In their study, Martins et al. (2014) have performed a continuous assessment of nurses’ emotions when facing their patients’ pain and distress. They report that nurses often identify with the pain and distress of patients and their family members [29]. In a study by Badger and Royse (2012), nurses were found to attempt to empathize with patients and their family members by attentively listening to them and trying to understand their experiences and concerns [30]. The results of another study demonstrate that if patients find caregivers unapproachable and unfriendly, they feel insecure and consider their behaviors as disrespectful [31]. Gallagher et al. (2008) report that failure to dedicate adequate time to talk to patients, lack of eye contact, and negligence make patients feel worthless and humiliated [32]. Likewise, Ebrahimi et al. (2012) report that nurses’ poor verbal and nonverbal communication skills weaken the emotional connection between nurses and patients, which consequently creates feelings of humiliation and neglect in the patients [22]. Failure to establish an effective relationship with patients gives patients the impression that their caregivers do not value them and have no respect for their dignity [33]. Thus, one of the major factors in maintaining patient dignity is the quality of the relationship of the medical staff, including nurses, with them [34]. Thus, by using effective communicative skills, the nurses in burns departments should try to understand their patients, treat them with respect for their human values, earn their trust and encourage them to cooperate, and dedicate time to understanding their various needs. Only then can the dignity of burn patients be preserved.

Based on the experiences of the interviewed participants, respect plays a significant role in maintaining the dignity of burn patients. The theme of showing respect consists of the following categories: respect for human equality, respect for autonomy, respect for beliefs and values, respect for sexual privacy, and avoidance of pity. To maintain the dignity of their burn patients, nurses should respect the patients’ beliefs and values, involve them in their treatment process, and respect their sexual privacy. Moreover, they should avoid pitying them. Similarly, several studies have emphasized the necessity of showing respect for the intrinsic value of humanity and the fact that dignity should not be a function of such factors as age, wealth, education, and severity of a patient’s illness [4, 15–35]. According to Baillie (2009), all human beings have equal rights to dignity, which should be acquired and cannot be taken away [36]. In their study, Matiti and Trorey (2008) mention that patients expect nurses to maintain their dignity, regardless of their social classes or health conditions [33]. The results of a study by Bagherian et al. (2019) in Iran show that cancer patients demand that their values should be respected. Patients’ experiences demonstrate that maintaining patient autonomy and equality in care is essential to respecting their ethical values and to preserving their dignity [17]. In their review of the available literature, the present researchers could not find any studies on the dignity of burn patients; however, it appears that the necessity of respecting patients’ equality and not differentiating between them as shown in the present study is rooted in the religious values of Iranians. Respect for the fact that all humans are equal in nature is one of the key components of burn patients’ dignity.

The participants of the present study also stated that, due to their prolonged treatment processes and various complications associated with their injuries, burn patients and their family members feel more valued if they can participate in the decision-making processes and treatment plans arranged for them. Pepastaro et al. (2016) report that patients’ dignity is maintained when patients are involved in the making of medical decision related to them [37]. Likewise, Baillie and Matiti (2013) conclude that observance of patient autonomy is a fundamental principle of patient-centered care, resulting in the preservation of patients’ dignity [38]. Even though the concept of burn patients’ dignity and the factors which affect it had not been studied before, it appears that preparing burn patients for participation in their clinical decision-making and giving them the feeling that they are involved in their treatment and care and have control over what happens to them play a key part in maintaining their dignity.

Based on the experience of the interviewed participants, attention to and respect for the beliefs and wishes of burn patients in Iran stand for respecting their dignity. Similarly, in other studies, showing respect for patients’ values is considered as an essential act to preserve their dignity. In a study, respecting patients’ values ​​and beliefs is reported as a component of professional patient-centered care [17]. Also, the concept of human dignity is dependent on cultural contexts. In all areas of healthcare, caregivers should respect patients’ values, be aware of their cultural orientations, and learn about their cultural perspectives on health and sickness [16]. The participants’ stressing the importance of showing respect for the patients’ values can be attributed to the religious beliefs of the patients and their families in the present study, all of whom were Shia Muslims. Islam dictates that all humans deserve to be respected. In other words, the tradition of respecting others in Iran may be rooted in the collectivism and cultural values of the Iranian people. In most Asian cultures, collectivism lies at the core of the common social belief system, and in a collectivist culture, an individual’s world view is mostly affected by the society, so individuals perceive themselves as entities attached to the society [39, 40]. Based on the experiences of the burn patients in the present study, showing respect for the sexual privacy of burn patients and receiving care from nurses of the same gender are important. The social norms in the cultural context of Iran dictate that individuals should be sensitive about keeping their bodies, especially their genitalia, covered and that Muslim men and women must avoid any physical contact with strangers of the opposite sex. Accordingly, in the Iranian culture, observance of same-gender care is essential to maintaining patients’ dignity.

According to the experiences of the caregivers, the dignity of burn patients may be undermined by expressions of undue pity from the members of treatment teams or other people nearby. The results of the conducted interviews showed that, sometimes, the members of treatment teams or burn patients’ companions unintentionally cause more pain and suffering to the patients, rather than soothing them, by pitying behaviors. Similarly, Bagherian et al. (2019) report that the dignity of patients is preserved when they are not pitied [17]. It seems that some caregivers do not have good communication skills and instead of conveying empathy only show that they feel sorry for their patients without suggesting any practical solutions. This behavior is interpreted as an expression of pity by patients and their companions. Based on the findings of the study, avoiding differentiating between patients, showing respect for patients’ autonomy, giving the patients the right to choose, engaging patients in their care process, and avoiding pitying behaviors are among the factors which pave the way for preserving burn patients’ dignity.

Comprehensive support, another theme of burn patients’ dignity, is comprised of the categories of pain relief, psychological support, and social support. From the participants’ point of view, the destructive and traumatic effects of burn injuries affect all aspects of the patients’ existence. Most of the caregivers reported pain as the most serious physical problem experienced by burn patients. Similarly, the results of another study show that all burn patients suffer from daily pains [41] and these experiences remain in their memory forever [42]. In this situation, alleviating the patients’ pain and meeting their other needs are the main responsibilities of nurses and are considered as the signs of respect for patients’ rights. Burn patients often need nurses to support them by applying proper pain management techniques [43]. Pain management is an ethical responsibility of caregivers and is known as an essential principle in nurses’ professional code of ethics [44]. However, the experiences of most burn patients indicate poor pain management on the part of caregivers [45]. As the findings of the study show, the nurses in burns departments should consider their patients’ physical needs, especially pain management, and take steps to remove those needs toward maintaining the patients’ dignity.

According to the results of the interviews with the caregivers, burn injuries not only affect the patients’ bodies, but also adversely affect their mental health, family members, family relationships, and social activities (e.g. participation in social activities, employment, and education). In some cases, divorce and other catastrophic life changes happen due to burn patients’ hospitalization because of their injuries. According to the experiences of the caregivers, the devastating nature of burn injuries and the ensuing complications damage the ego of burn victims. The results of a study conducted in Iran show that burn survivors experience threats to all dimensions of their “self” in the form of disturbance to their feelings, cognition, sense of identity, and behaviors. All these emotional-cognitive disturbances can cause burn survivors to experience “self-disruption.” In addition, most of the participants in a study have reported that they were able to overcome their hopelessness through their belief in God and receiving support from the people around them [46]. The findings of the present study show that burn patients are at the risk of losing their power to control and manage their inner, inter-personal, and outside worlds following the physical, mental, and social tensions caused by their injuries. These conditions potentially threaten burn patients’ dignity. Thus, nurses are expected to take measures to protect their burn patients’ self-discipline and respect their identity toward maintaining the patients’ dignity.

Research suggests that the dignity of hospitalized patients can be threatened by certain factors, including patients’ inability to assume their previous roles in life, lack of support from friends and the medical personnel, and uncertainty about the future [8]. In a study by Bagheri et al. (2018), disregard for patient participation and failure to support patients are reported to cause patients’ loss of dignity [47]. By studying the stigma of having burn injuries from the perspective of burn victims’ families at the time of their patients’ hospital discharge, Rossi et al. (2005) conclude that burn patients’ families are worried about and ashamed of the society’s attitude to their injured family members [48]. Several studies conducted in different countries also suggest that family support can facilitate burn patients’ adaptation, improve their quality of life, promote their mental rehabilitation, and help them cope with their depression [4, 49]. However, not much research is currently available on the role of families and their support in the preservation of patients’ dignity [16]. In the Iranian culture, the family is the main source of emotional support for patients; therefore, the presence of family members and relatives at the side of their patients is known as a part of patients’ regular care and their families’ social and religious values. In this culture, a holistic healthcare system dictates that in the hospital environment, professional caregivers should tend to the needs of patients as well as their families [49]. Holistic care acknowledges human dignity, regards patients as one with their environment, and considers the body, mind, and soul of patients. Furthermore, recognizing the role of patients in the treatment process, allowing patients to participate in their care, and encouraging patients to practice self-care are some of the other aspects of holistic care which result in the preservation of patients’ dignity and autonomy [50]. One of the signs of respect for burn patients’ dignity during their care is providing them with a kind of care in which all the existential aspects of the patients are taken into account. Thus, comprehensive evaluations of burn patients, planning care according to the results of the evaluations and the patients’ demands, and providing holistic care are among the primary responsibilities of nurses by which they can maintain their patients’ dignity. The complicated nature of caring for burn patients obliges nurses to have a good knowledge of nursing care techniques as well as psychosocial skills to be able to support their patients and preserve their dignity.

In conclusion, the findings of the present study show that the dignity of burn patients is a multi-faceted phenomenon which mostly depends on the cultural context of patients and can be preserved via empathic communication with patients, respecting them and giving them comprehensive support. In addition, it appears that empathic communication can lay the foundation for providing comprehensive support, and showing respect can guarantee the continuation of effective communication and comprehensive support.

One of the limitations of the present study is that a limited number of family caregivers and burn patients were interviewed at the data collection stage. Also, the viewpoints of other members of treatment teams were not taken into account and the data were exclusively collected through individual interviews in the context of an educational burn hospital in south of Iran. Therefore, the researchers suggest that, to collect richer data, future studies should use larger samples of family caregivers and burn patients selected from various environments and include the experiences of other members of treatment teams, including doctors, physiotherapists, psychologists, and social workers. Also, it is suggested that future studies employ other methods of data collection as well, including observation and focus group interviews.

## Conclusion

Showing respect for patients’ rights and dignity has been emphasized as one of the ethical responsibilities of professional caregivers. The dignity of burn patients is potentially at risk due to numerous physical, psychological, and social factors. The results of the present study confirm that preserving the dignity of burn patients produces positive outcomes. For the dignity of burn patients to be preserved, the patients should be respected and provided with comprehensive care in an effective communication. The results of this study can also help healthcare managers and policy-makers to create a supportive environment in which burn patients’ dignity is effectively preserved. In the present study, empathic communication, dedicating time to listening to patients, showing respect for patients’ beliefs and values, providing care free of discrimination, and giving comprehensive support were found to be effective ways to maintain the dignity of burn patients.

## Supplementary Information

**Additional file 1.** Interview Guide.

## Data Availability

The dataset generated and/or analysed during the current study are not publicly available due to promises of participant anonymity and confidentiality but are available from the corresponding author on reasonable request.

## Abbreviations

PhD: Doctor of Philosophy
ICU: Intensive Care Unit
