# Supplementary material for: The dignity of burn patients: a qualitative descriptive study of nurses, family caregivers, and patients
Source: BMC Nurs. 2021 Oct 22;20:205. doi: 10.1186/s12912-021-00725-w (PMC8539828; doi:10.1186/s12912-021-00725-w)
Supplement: Supplementary file 1 — Additional file 1. Interview Guide. [file 12912_2021_725_MOESM1_ESM.docx]

**General question (Nurses/** **Family caregivers/** **Burns patients)**

What does the concept of burn patients' dignity mean to you?

**Specific questions (Nurses)**

What experiences have you had related to the dignity of burn patients?

Explain your experiences regarding dignity of burn patients during a work shift?

How do you feel after respecting or ignoring a patient's dignity?

When you talk about dignity of burn patients, what comes to your mind?

**Specific questions (Family caregivers)**

What are your experiences of the dignity of burn patients during hospitalization?

Please mention one example of the dignified care services that your patient has received?

How do you feel when your patient receives dignified care?

Describe your feelings when your patient does not receive dignified care?

**Specific questions (Burns patients)**

What are your experiences of having had your dignity preserved during your stay in the hospital?

What conditions would threaten your dignity?

How do you feel when your dignity is maintained or ignored?

According to your experiences, how do you define dignity of burn patients?
